# Dr. George Gregory Carroll

**Published:** 1905-11

**Authors:** 


Dr. GEORGE GREGORY CARROLL, of Rochester, died at his home September 25, 1905, aged 62 years. He graduated from the medical department of the University of Buffalo in 1870 and was a respected member of the profession in the city of his residence.

